# 2023 ISCB accomplishments by a senior scientist award: Mark Gerstein

**DOI:** 10.1093/bioinformatics/btad316

**Published:** 2023-06-30

**Authors:** Christiana N Fogg, Diane E Kovats, Martin Vingron

**Affiliations:** Kensington, MD, United States; International Society for Computational Biology, Leesburg, VA, USA; International Society for Computational Biology, Leesburg, VA, USA; Max-Planck-Institut fuer molekulare Genetik, Germany

ISCB recognizes the outstanding contributions by a leader in the fields of computational biology and bioinformatics annually with the Accomplishments by a Senior Scientist Award. This award is the highest recognition conferred by ISCB to a scientist who has made notable research, education, and service contributions to the field and to ISCB. Mark Gerstein, Albert L. Williams Professor of Biomedical Informatics, Molecular Biophysics and Biochemistry, Computer Science and Statistics and Data Science at Yale University, New Haven, CT, is the 2023 recipient of the ISCB Accomplishments by a Senior Scientist Award. He will be presented his award and deliver a keynote address at the 2023 ISMB/ECCB conference in Lyon, France.


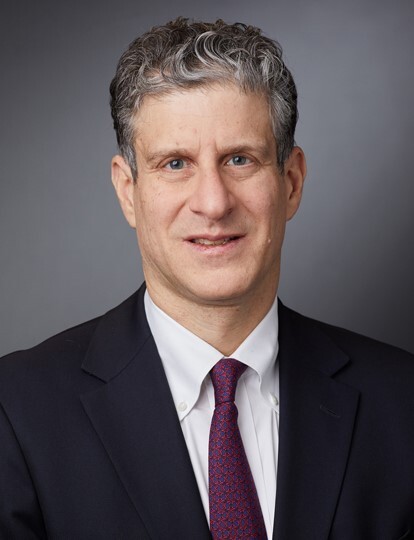
 *Mark Gerstein, Yale University. Photo credit: Robert Lisak*.

## Mark Gerstein: from hacker to architect of computational biology

Mark Gerstein was born in New York City and recalled a childhood where his interests in science and mathematics were nurtured and encouraged. As a young child, he fondly remembers becoming engrossed in a science project constructing a model of the DNA double helix, foreshadowing his future interest in biological macromolecules. Gerstein’s intellectual curiosity led him to double major in physics and the history of science at Harvard College. Although he enjoyed physics and was curious about the nascent field of computer science, Gerstein ultimately wanted to pursue a PhD in a growth area of science. He recalled, “I really wanted to look at the confluence of biological science and computation.” This was at a time when the structures of large macromolecules were just beginning to be resolved using computers. Gerstein was encouraged to pursue these interests at Cambridge University through his conversations with Martin Karplus and Don Wiley at Harvard. Their recommendation connected to his ongoing fascination with Cambridge given its storied place in scientific history, including Watson and Crick’s discovery of the DNA double helix and the development of the theory of computation by Cambridge alumnus Alan Turing. Gerstein was given a Herschel-Smith Scholarship to pursue his PhD at the Chemistry Department and the Medical Research Council (MRC) in Cambridge, during which time he worked with computational chemist Ruth Lynden-Bell and protein biophysicist (and 2015 ISCB Accomplishments by a Senior Scientist Award Winner) Cyrus Chothia. His project involved developing computer simulations of liquids, including water, and their interaction with proteins, which laid the foundation for his future postdoctoral studies. Gerstein also came to appreciate that his time at MRC brought him into contact with many gifted scientists, including future Nobel Prize winners such as Venkatraman Ramakrishnan and Richard Henderson.

Gerstein moved on to postdoctoral studies in 1993 under the mentorship of future Nobel laureate Michael Levitt at Stanford University, where he used his newly minted skills in modeling to study macromolecular geometry and simulate water surrounding proteins. Gerstein tapped into his computer hacking passion and brought LINUX to Levitt’s lab. He recalled, “Not only was Levitt a gifted scientist, but he was also a computer hacker.” Levitt’s mentorship helped Gerstein realize that working at the interface of biology and computation was an exciting and viable career path. He also got to know Russ Altman during his post-doc and ultimately attended the first ISMB in 1993. Gerstein said, “I started to see there were a lot of things you could do with these large biological datasets.”

Gerstein’s productive postdoc years were critical to launching his career as an independent investigator. He was hired in 1997 as an assistant professor at Yale University. He was one of the first computational biology faculty members hired by a large research university, and he had anticipated building a lab that studied macromolecular modeling. Early projects included simulation and classification of protein motions using a database framework. He was also intrigued by the emerging area of genome sequencing. This led Gerstein to study structural genomics and build up his research program with collaborators at numerous institutions. After receiving tenure, Gerstein became interested in human genome annotation and later became deeply involved with ENCODE and related large-scale projects, such as psychENCODE. These interests evolved into several high-impact publications demonstrating that multi-omics data can be reframed as control networks and can be compared to networks in other contexts—for instance, in social relations. These studies have been critical to identifying regulatory sites in genomes and in finding pseudogenes and improving our understanding of genome evolution. Gerstein and his lab were also involved in the 1000 Genomes Project and applied concepts developed from this work to develop tools for more accurate variant interpretation with respect to risks for cancer or neuropsychiatric diseases. His lab now examines many aspects of data science, including the large-scale integration of genomic and phenotypic data, collected by biosensors and images, and the attendant privacy concerns.

Gerstein considers his efforts to develop undergraduate and graduate computational biology programs at Yale to be one of his most lasting contributions to the field. He said, “Computational biology is important, and part of making it a field is education.” He has taught his introductory undergraduate computational biology class since 1998, when it began as a 10-student course called “Genomics and Bioinformatics.” He has made every set of lecture slides available online, toward his mission to educate the world more broadly about computational biology. The course in its current form, called “Biomedical Data Science,” provides students with a range of immersive experiences, including the culminating project in which students analyze a chromosome from the science writer Carl Zimmer’s genome and present their findings to Zimmer in person. Gerstein was also integral to co-founding the Computational Biology Graduate Program at Yale with his colleague Perry Miller over 20 years ago and has watched many of the program’s graduates move into their own faculty positions. Gerstein himself has mentored more than 125 trainees, of which nearly 40 have gone on to start their own labs. Gerstein sees his work as a mentor within and beyond the lab as integral to advancing the field of computational biology. His prestigious publication record reflects the efforts of Gerstein and his trainees, including over 650 publications and 189 000 citations. He is an ISCB and AAAS Fellow and has served on numerous editorial boards, working groups and committees. Gerstein is also a frequent contributor to Op-Ed columns, using his voice to communicate the nuances of data science in various contexts to a wider audience.

Gerstein still gets very excited about computational biology and considers the field to hold a unique place among the data sciences. He said, “In the future, computational biology has an important role for how we go forward with data science. Now people are seduced by big data, but computational biology is a bridge between big data, physical modeling, and a mechanistic description of how biology is actually carried out on a molecular scale.” The same cannot be said yet for big data applications in political or social sciences, and computational biology uniquely feeds human curiosity as to how living things work.

Gerstein is deeply honored to be recognized with the 2023 ISCB Senior Scientist Accomplishment Award, as it is a recognition of his contributions from his peers, and it serves as further validation that the field of computational biology has matured to stand alone and guide the future of data science.

